# Serpentinization: Connecting Geochemistry, Ancient Metabolism and Industrial Hydrogenation

**DOI:** 10.3390/life8040041

**Published:** 2018-09-22

**Authors:** Martina Preiner, Joana C. Xavier, Filipa L. Sousa, Verena Zimorski, Anna Neubeck, Susan Q. Lang, H. Chris Greenwell, Karl Kleinermanns, Harun Tüysüz, Tom M. McCollom, Nils G. Holm, William F. Martin

**Affiliations:** 1 Institute of Molecular Evolution, University of Düsseldorf, 40225 Düsseldorf, Germany; xavier@hhu.de (J.C.X.); zimorski@hhu.de (V.Z.); 2 Division of Archaea Biology and Ecogenomics, Department of Ecogenomics and Systems Biology, University of Vienna, Althanstrasse 14 UZA I, 1090 Vienna, Austria; filipa.sousa@univie.ac.at; 3 Department of Earth Sciences, Palaeobiology, Uppsala University, Geocentrum, Villavägen 16, SE-752 36 Uppsala, Sweden; anna.neubeck@geo.su.se; 4 School of the Earth, Ocean, and Environment, University of South Carolina, 701 Sumter St. EWS 401, Columbia, SC 29208, USA; slang@geol.sc.edu; 5 Department of Earth Sciences, Durham University, South Road, DH1 3LE Durham, UK; chris.greenwell@durham.ac.uk; 6 Institute for Physical Chemistry, University of Düsseldorf, 40225 Düsseldorf, Germany; shangrila1@icloud.com; 7 Max-Planck-Institut für Kohlenforschung, Kaiser-Wilhelm-Platz 1, 45470 Mülheim an der Ruhr, Germany; tueysuez@mpi-muelheim.mpg.de; 8 Laboratory for Atmospheric and Space Physics, University of Colorado, Boulder, CO 80309, USA; tom.mccollom@lasp.colorado.edu; 9 Department of Geological Sciences, Stockholm University, SE-106 91 Stockholm, Sweden; Nils.Holm@geo.su.se

**Keywords:** rock–water–carbon interactions, origin of life, carbides, iron sulfur, early metabolism

## Abstract

Rock–water–carbon interactions germane to serpentinization in hydrothermal vents have occurred for over 4 billion years, ever since there was liquid water on Earth. Serpentinization converts iron(II) containing minerals and water to magnetite (Fe_3_O_4_) plus H_2_. The hydrogen can generate native metals such as awaruite (Ni_3_Fe), a common serpentinization product. Awaruite catalyzes the synthesis of methane from H_2_ and CO_2_ under hydrothermal conditions. Native iron and nickel catalyze the synthesis of formate, methanol, acetate, and pyruvate—intermediates of the acetyl-CoA pathway, the most ancient pathway of CO_2_ fixation. Carbon monoxide dehydrogenase (CODH) is central to the pathway and employs Ni^0^ in its catalytic mechanism. CODH has been conserved during 4 billion years of evolution as a relic of the natural CO_2_-reducing catalyst at the onset of biochemistry. The carbide-containing active site of nitrogenase—the only enzyme on Earth that reduces N_2_—is probably also a relic, a biological reconstruction of the naturally occurring inorganic catalyst that generated primordial organic nitrogen. Serpentinization generates Fe_3_O_4_ and H_2_, the catalyst and reductant for industrial CO_2_ hydrogenation and for N_2_ reduction via the Haber–Bosch process. In both industrial processes, an Fe_3_O_4_ catalyst is matured via H_2_-dependent reduction to generate Fe_5_C_2_ and Fe_2_N respectively. Whether serpentinization entails similar catalyst maturation is not known. We suggest that at the onset of life, essential reactions leading to reduced carbon and reduced nitrogen occurred with catalysts that were synthesized during the serpentinization process, connecting the chemistry of life and Earth to industrial chemistry in unexpected ways.

## 1. Abiotic Chemical Synthesis at Hydrothermal Vents

Since their discovery, hydrothermal vents have been of interest in thoughts about the origin of life [1,2]. They are relevant to origins for a number of reasons. From the standpoint of thermodynamics, hydrothermal systems harbor chemical reactions that are continuously far from equilibrium, a property they share with life [3,4,5], and they harbor gradients: Temperature gradients, pH gradients, and redox gradients [2,6]. Today, those gradients are most pronounced at the vent ocean interface, where vent effluent emerges into sea water and forms hydrothermal mounds [6,7]. But life is—to our knowledge—not arising anew today. Rather life emerged once. We can say that because all life forms we know (the only ones that demand an explanation) share the same genetic code, this can only be reasonably explained by common ancestry [8]. Carbon isotopes with signatures typical for life that appear in rock 3.95–3.8 Ga of age [9,10] provide the currently known date for the emergence of life.

Though views on the origin of life are traditionally marked by debate, everyone agrees that energy was important, because without energy uptake and release, no chemical reactions can take place, and if no chemical reactions take place, life forms can neither arise nor multiply. Chemical reactions require the flow of energy in order to be initiated and proceed. The main sources of energy at origins that are currently discussed in the literature are *UV* light [11,12], lightning that generates nitric oxides as oxidants [13], kinetic energy of meteorite impacts [14,15], hydrothermally generated ion gradients [16,17,18], and chemical energy in the form of the H_2_–CO_2_ redox couple [19,20,21,22,23,24,25].

This paper will focus on H_2_-dependent CO_2_ reduction in hydrothermal systems and its possible significance at origins. We will discuss H_2_ synthesis in hydrothermal systems via serpentinization, H_2_-dependent organic synthesis at modern vents, and H_2_-dependent reduction of inorganic compounds in the crust to generate catalysts (native metals and carbides) that are used in the laboratory and in industry for H_2_-dependent reduction of CO_2_ and N_2_. Catalysts are important because they increase reaction rates by lowering the activation energy. They also have a drastic effect on the type of products formed because in thermodynamically controlled reactions the most stable products accumulate, whereas in kinetically controlled reactions the most rapidly formed products accumulate. The nature of the most rapidly formed products is usually governed by the chemical and physical properties of the catalyst.

We will outline clear links between vents and well-studied existing life forms. Modern anaerobic autotrophs that live from the H_2_–CO_2_ couple, acetogens and methanogens, have a conserved native metal (Ni^0^) in their most central CO_2_ reducing enzyme—carbon monoxide dehydrogenase (CODH) [26,27]—and a carbide in the only enzyme that introduces N_2_ into biology, nitrogenase [28,29]. At the same time, H_2_-dependent reduction of inorganic compounds uncovers interesting links between vents and important industrial processes, because serpentinization yields not only H_2_, but also Fe_3_O_4_, which is the starting catalyst for CO_2_ hydrogenation to synthetic gasoline [30] and for N_2_ reduction to NH_3_ through the Haber–Bosch process [31,32].

That the chemical conditions, minerals and metallic compounds of hydrothermal vents could serve as sites of chemical synthesis of organic compounds on the early Earth is hardly a new idea [3,33,34]). Shock and Schulte [35] investigated the thermodynamics of hydrothermal systems, predicting nearly complete conversion of inorganic CO_2_ to organic compounds under some conditions, emphasizing that organic compounds should even be more stable than mixtures of their precursors H_2_ and CO_2_ under a variety of conditions. Amend and Shock [36] showed that the synthesis of amino acids was thermodynamically favorable under hydrothermal vent conditions. Amend and McCollom [37] explored the thermodynamics of low temperature vents similar to Lost City (low temperature, slight pH gradients between effluent and sea water), and found that synthesis of organic compounds in the composition and stoichiometry of biomass from inorganic precursors is thermodynamically favorable (−1016 to −628 Joules per cell) at 50 to 100 °C. Clearly, there is substantial potential for organic synthesis at today’s hydrothermal vents and likely more so in vents on the early Earth. If life really started 3.8–3.95 billion years ago [9], we need to consider the state of the very early Earth, the setting within which the first hydrothermal systems formed.

## 2. The Early Earth: Magma, then Crust, then Oceans

The early Earth was molten following the moon-forming impact roughly 4.4 billion years ago, at the latest [38,39]. With temperatures on the molten Earth around > 1200 °C, carbon that had been brought to Earth by accretion was converted to CO_2_, which was outgassed into the atmosphere [38,40], with a portion of CO_2_ remaining in magma oceans [41]. Magma oceans also retained little water, which was predominantly converted to atmospheric steam. By about 4.4 Ga the magma ocean had cooled [42] and by about 4.2 Ga there was liquid water on Earth [38,39], some condensed from the atmosphere and some delivered later by comets. By around 4 Ga the late heavy bombardment had ended [42,43]. By 3.95 Ga a carbon isotopic signature compatible with that produced by the acetyl-CoA pathway had appeared [9].

As it relates to hydrothermal vents and organic synthesis, the relevant sequence of events starts from CO_2_ as a result of magma oceans and minor amounts of mantle CO_2_. In the molten state, the densest material of the early Earth—metals in the elemental state—was drawn to the planet’s center where it remains to this day as the core (mainly 85% Fe and 5% Ni). This process of differentiation (gravitational metal migration to the core) did not completely separate heavy material from light, because some light elements also exist in the core, such as Si, S, and C, estimated at 6%, 2%, and 0.2% respectively [44]. Lighter material consisting mainly of silicates (iron, magnesium, and aluminum silicates), possibly with residual metals, was displaced to the surface accordingly, where it formed the primordial mantle and crust. The crust started as magma and, thus, had a very low water content [38]. As the crust cooled, water eventually condensed over the Earth’s surface to form oceans of liquid water [38,40,45,46].

**Figure 1:**
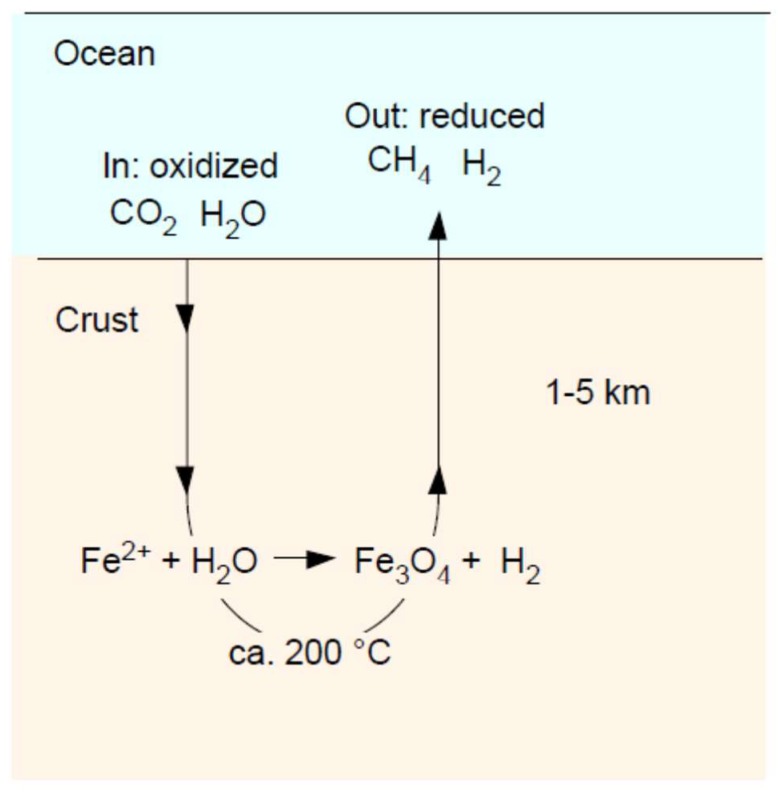
Schematic representation of serpentinization in a hydrothermal vent. See text and [5,48,49,50,51,52].

As liquid oceans formed, gravity pulled water into cracks of the steadily cooling crust. Water in the crust became heated and resurfaced, creating convective currents. The primordial ocean was about twice as deep as today’s because the modern crust and mantle bind about one ocean volume of water [40,47], which was originally in the ocean before rock–water interactions in the primordial crust commenced. The CO_2_ content of the primordial atmosphere was perhaps 100–1000 times higher [40] than today’s, and very large amounts of CO_2_ were, thus, dissolved in the ocean. Convective water currents through the very dry iron magnesium silicate crust led to rock–water interactions, initiating a process called serpentinization, as sketched in Figure 1.

## 3. Serpentinization: Rock–Water Interactions

Serpentinization occurs when ultramafic rocks, enriched in the minerals olivine and orthopyroxene, react with water and are converted to rocks containing a suite of minerals dominated by serpentine. A significant by-product of the reaction is H_2_. In general terms, the process can be summarized by the general reaction:

Olivine ± orthopyroxene + H_2_O → serpentine ± brucite + magnetite + H_2_. (1)

In somewhat more precise terms, the reaction can be expressed as:

(2) $$\begin{array}{c} {\mathrm{Mg}}_{1.8}{\mathrm{Fe}}_{0.2}{\mathrm{SiO}}_{4}\left[\mathrm{olivine}\right]\;+\;{\mathrm{Mg}}_{1.8}{\mathrm{Fe}}_{0.2}{\mathrm{Si}}_{2}O_{6}\left[\mathrm{orthopyroxene}\right]\;+\;{\mathrm{wH}}_{2}O\\ 0.5{\left({\mathrm{Mg},\mathrm{Fe}}^{2+}{,\mathrm{Fe}}^{3+}\right)}_{3}{\left({\mathrm{Si},\mathrm{Fe}}^{3+}\right)}_{2}O_{5}{\left(\mathrm{OH}\right)}_{4}\left[\mathrm{serpentine}\right]\;+x\left({\mathrm{Mg},\mathrm{Fe}}^{2+}\right){\left(\mathrm{OH}\right)}_{2}\left[\mathrm{brucite}\right]\;+y{\mathrm{Fe}}_{3}O_{4}\left[\mathrm{magnetite}\right]\;+zH_{2} \end{array}$$

where the stoichiometric coefficients *w*, *x*, *y*, and *z* are variable and depend on a number of factors including temperature and the relative proportions of olivine and orthopyroxene in the reacting rock [48,49]. The production of H_2_ during serpentinization results from the oxidation of ferrous iron (Fe^2+^) from the reactant minerals to ferric iron (Fe^3+^) in the products through reaction with water, liberating H_2_. This process can be expressed as:

2(Fe^2+^O) + H_2_O → (Fe^3+^_2_O_3_) + H_2_ (3)

where (Fe^2+^O) and (Fe^3+^_2_O_3_) represent components of reacting rocks and product minerals, respectively. Magnetite is often the major product of Fe^2+^ oxidation during serpentinization. In some serpentinizing systems, sufficient amounts of H_2_ accumulate to reduce Fe^2+^ and Ni^2+^ (a trace component of olivine), converting them to native metal alloys such as awaruite (Ni_3_Fe) [53,54].

(Fe^2+^O) + 3(Ni^2+^O) + 4H_2_ → Ni_3_Fe [awaruite] + 4H_2_O (4)

where (Fe^2+^O) and (Ni^2+^O) again represent components of the reacting rock. The essence of serpentinization is, ultimately, that Fe^2+^ is oxidized by water, water is consumed by the reaction, the oxidized Fe^2+^ and the newly formed oxygen remain as magnetite (Fe_3_O_4_), the water is reduced to H_2_, and the vent fluid becomes alkaline (pH 9–11) [6] due to hydroxides being generated. Notably, magnetite, Fe_3_O_4_, and H_2_ are the starting materials for industrial catalysts used for N_2_ reduction via the Haber–Bosch process, to which we will return in a later section. Liquid gasoline synthesis from H_2_ and CO_2_, according to new findings [30] is also efficiently and specifically (up to 22% CO_2_ conversion and 78% of products) catalyzed with magnetite. In industrial CO_2_ reduction, iron catalysts are often the choice for high-temperature processes.

However, if we consider serpentinization as a source of energy and electrons for organic synthesis at the origin of life [5,50,51,52] or as a source of energy and electrons for the first ecosystems on Earth [38,55,56], we have to extrapolate back from modern systems. Importantly, only ultramafic (silica-poor) rocks can undergo serpentinization. The ultramafic rocks that are serpentinized in today’s ocean lithosphere represent chunks of the mantle that are tectonically uplifted to shallow environments where serpentinization happens owing to lower temperatures [57,58]. Since the composition of the mantle does not appear to have changed significantly over time, primordial serpentinization should not have been fundamentally different from the modern process. Furthermore, a possibly thinner crust and the eruption of extrusive ultramafic volcanic rocks called komatiites because of higher mantle temperatures would have made this process more widespread on the early Earth than it currently is [35]. The primordial crust contained minor amounts of residual Fe^0^ and Ni^0^ from differentiation, and the ocean water circulating through the crust contained much more CO_2_ than present day.

On the modern seafloor, fluids discharged from serpentinizing systems contain up to 16 mmol H_2_/kg [59,60]. Laboratory simulations of serpentinization generate comparable and higher H_2_ concentrations [61,62,63]. Hydrothermal fluids from serpentine-hosted seafloor systems are also commonly enriched in CH_4_, up to several mmol/kg [59,60]. The exact temperature and pressure in natural serpentinizing systems are not known, serpentinization experiments in simulated hydrothermal systems show good results from 400 °C down to nearly ambient temperatures [64,65].

## 4. Serpentinization: Awaruite and Carbon

Some serpentinization loci are naturally richer in nickel and iron minerals than others, leading to alloys containing compounds like taenite (NiFe, 25–40% Ni), kamacite (NiFe, ca. 7% Ni), or awaruite [65,66]. The Ni–Fe alloys produced in serpentinizing systems are primarily awaruite, which can have a somewhat narrow range of composition from Ni_2_Fe to Ni_3_Fe (Ni_2-3_Fe). Awaruite is a minor but fairly widespread component of serpentinized rocks [53,66,67]. Its mechanism of synthesis and/or deposition are not known in detail, but it is known to be generated by serpentinization because it occurs in hydrothermally altered serpentinite rocks [52,68]. It is thought to arise from the H_2_-dependent reduction of Fe^2+^ and Ni^2+^ containing minerals during the serpentinization process [53,66]. In a laboratory simulation of serpentinization, McCollom [68] reported the laboratory synthesis of awaruite from Ni-containing olivine under conditions that generated ca. 60 mmol/kg H_2_. In the temperature range 200–400 °C, high H_2_ activities and low H_2_S activities favor awaruite formation [53]. Foustoukos et al. [54] report that awaruite can be formed at H_2_S activities of ca. 1 mmol/kg and at H_2_ activities of <100 mmol/kg, in line with findings from laboratory awaruite synthesis [68].

Serpentinization in submarine hydrothermal systems has been around since there was water on Earth [55]. Contingent upon a heat source and cooling rates at the ridge axis, the reaction runs continuously until the host rocks are completely serpentinized, or the supply of water is exhausted. Lost City is at least 30,000 years old [69] and possibly 100,000 years old [70], indicating that these systems can be fairly long-lived. Lost City effluent contains 1–2 mmol/kg methane [71], with formate being the second most prevalent carbon species [72]. Like methane, the isotopic composition of Lost City formate indicates an abiotic origin [56,72]. Smaller amounts of ethane, propane, and butane are also observed in a serpentinizing environment and attributed to abiological synthesis [71,73,74,75].

During the lifespan of a serpentinizing vent, the rock reacts and its composition changes, altering the redox state and chemical composition of its surfaces, an aspect that might be relevant in terms of catalyst formation during serpentinization.

A study by Horita and Berndt [76] illustrates the principle. In laboratory experiments to better understand the presence of methane in hydrothermal effluent, they examined the reduction of CO_2_ with H_2_ to CH_4_ using awaruite as the catalyst. At temperatures from 200–400 °C, pressure at 50 bar (5 MPa), and H_2_ activities around 200 mmol/kg, they obtained 1–10 mmol/kg CH_4_, often with >50% conversion rates of CO_2_ added. The point is that awaruite (the catalyst) is not a pre-existing component of the rocks that host hydrothermal systems. Awaruite is first synthesized on site by the serpentinization process, and once formed it can then catalyze the H_2_-dependent reduction of CO_2_.

The study of Horita and Berndt [76] points to the significance of native metals in rock–water–carbon interactions more generally. Heinen and Lauwers [77] investigated the ability of Fe^0^ to catalyze CO_2_ reduction in the presence of H_2_S to obtain thiols in an early evolution context. They obtained modest thiol yields (nmol), but they only analyzed volatile S containing compounds.

In the laboratory, good rates of CO_2_ conversion have been reported under hydrothermal conditions using native metals as catalysts and reductants. Guan et al. [78] showed that Fe^0^ in the presence of potassium, copper and aluminum will reduce CO_2_ to CH_4_, C_3_H_8_, CH_3_OH, and C_2_H_5_OH in the 10–70 µM range in 20 h at room temperature. He et al. [79] reported a reduction of CO_2_ to formate and acetate in the 1–10 mM range using nanoparticular Fe^0^ at 80–200 °C for 5 to 200 h. Varma et al. [80] used Fe, Ni, Mo, Co, and W at temperatures between 30–100 °C, all with some success, but the best yields (10–200 µM concentrations of reduced carbon compounds) were observed with Fe^0^ in presence of potassium salts. Importantly, the reduced carbon products observed by Guan et al. [78], He et al. [79], and Varma et al. [78] are compounds that also occur as intermediates and end products in the metabolism of organisms, such as acetogenic bacteria and methanogenic archaea [81,82], that live on H_2_ and CO_2_ as the substrates for their carbon and energy metabolism: Formate, methyl moieties, and acetate. Varma et al. [80] even reported the synthesis of pyruvate at temperatures of 100 °C and below.

In terms of microbial physiology, Varma et al.’s [80] findings are very significant because the reduction of CO_2_ to pyruvate—presumably via formate, methyl groups, and acetyl groups—exactly mirrors the reaction sequence in the acetyl-CoA pathway, the pathway of carbon and energy metabolism in organisms that live from the reduction of CO_2_ with H_2_ [83,84]. Stated another way, when CO_2_ and native metals react in water overnight under strictly anaerobic conditions, the core reaction sequence of the most ancient pathway of microbial carbon and energy metabolism, the acetyl-CoA pathway [24,83,85,86], which operates in the most ancient microbial lineages [87,88] unfolds in a series of spontaneous non-enzymatic reactions in the presence of water. The pressures employed by Varma et al. [80] do not preclude, however, the existence of a gas phase. At depths of several km, very high gas activities can be attained, but the pressure (hundreds of bars) would seem to be too high for a gas phase. However, Früh-Green et al. [89] reported gas bubbles arising during drilling around vents at the Atlantis Massif at a depth of 1,140 m.

Noteworthy in the study of Varma et al. [80] is that the products of CO_2_ reduction appear to be synthesized and bound on the surface of the metals, such that they had to be cleaved by alkaline hydrolysis to be assayed. It is not clear whether the products were bound to the Fe particles via C–Fe bonds or C–O–Fe bonds, because there was no surface analysis so far, but alkaline hydrolysis was required to obtain the soluble products. In the studies of He et al. [79] and Guan et al. [78] some fraction of the reduced carbon products was probably discarded, bound to metal surfaces. Reaction mechanisms for CO_2_ reduction have been proposed by He et al. [79] and Varma et al. [80] but the exact role of H_2_ and Fe^0^ in CO_2_ reduction is not yet clear. Heinen and Lauwers [77] showed that under anaerobic conditions, Fe^0^ and H_2_O readily generate H_2_ which, in the presence of the metal, readily reduces CO_2_. Guan et al. [78] reported that roughly 0.3 mol H_2_ was generated per mol Fe^0^.

Awaruite was also reported to catalyze the reduction of N_2_ with H_2_, but at very low rates just above background [90]. Under simulated deep crust conditions comparable to the Haber–Bosch process (>300 °C), but without exogenous H_2_, the reduction of N_2_ to NH_3_ on native iron was reported [91], as were low conversion rates (~0.1%) of N_2_ to NH_3_ with H_2_S in the presence of FeS at ambient pressure and 90 °C [92]. On the scales of submarine crust volume and geological time, a constant supply of small amounts of reduced nitrogen, or activated nitrogen species on metal catalyst surfaces, could easily be sufficient to underpin prebiotic synthesis of nitrogenous carbon compounds [93].

## 5. Serpentinization: Methane

Distinctive C and H isotope signatures and other evidence indicate that the methane discharged from many serpentinizing systems has an abiotic origin [71,74,75]. Presumably, this methane is produced as the H_2_ produced by serpentinization reacts with CO_2_:

CO_2_ + 4 H_2_ → CH_4_ + 2H_2_O (5)

Although exactly where in the system and how it is produced remains a matter of ongoing debate [74,75,94,95]. In the absence of catalysis, reduction of dissolved CO_2_ is extremely slow even at temperatures up to 350 °C [33,68,96]. However, when suitable catalysts such as awaruite are available, or when CO_2_ and H_2_ are present in a gas phase, the reaction can proceed much more readily [22,33,75,76]. It is this kinetic inhibition that allows CO_2_ and H_2_ to remain in disequilibrium and be exploited as an energy source by methanogenic organisms in hydrothermal environments. Reduction of dissolved CO_2_ to formate proceeds readily under simulated hydrothermal conditions and, as mentioned above, formate is observed in modern hydrothermal vent effluent, although typically at lower concentrations than methane [72,74,97].

Today, the formation of abiotic methane is important as a substrate for methanotrophic organisms in an oxidizing environment. In an origin of life context, methane itself is probably not of central interest, because of its very strong C–H bond. At great depths in the oceanic crust and in the upper mantle dominated by the fayalite-magnetite-quartz (FMQ) redox buffer, CO_2_ is the dominant carbon species at stable equilibrium [20]. At temperatures above about 350 °C, equilibrium between CO_2_ and CH_4_ favors CO_2_ even at elevated H_2_ concentrations [5,20]. However, with decreasing temperature, the equilibrium shifts to favor CH_4_, so that at temperatures below ~350 °C reaction of CO_2_ with H_2_ to form CH_4_ is thermodynamically favored. If the activation energy is too high and kinetic hindrances arise that prevent equilibrium conditions from being established, the organic chemistry will be locked up in different organic metastable compounds such as carboxylic acids. If nitrogen is present, even amino acids and nitrogen bases may be formed. Therefore, it is the interrupted transition from CO_2_ to CH_4_ that has the potential to create prebiotic constituents of life processes in the ocean floor and not CH_4_ itself. Organic chemists have often rejected igneous environments as a likely site for the origin of life because CO_2_ is a common component of fluids and gases (because of kinetic hindrances). Oparin [98], for instance, claims that ‘carbon dioxide is not the beginning but the end of life’—a statement that is, of course, only true in an oxidizing environment.

## 6. Serpentinization: Magnetite (Fe_3_O_4_)

**Figure 2:**
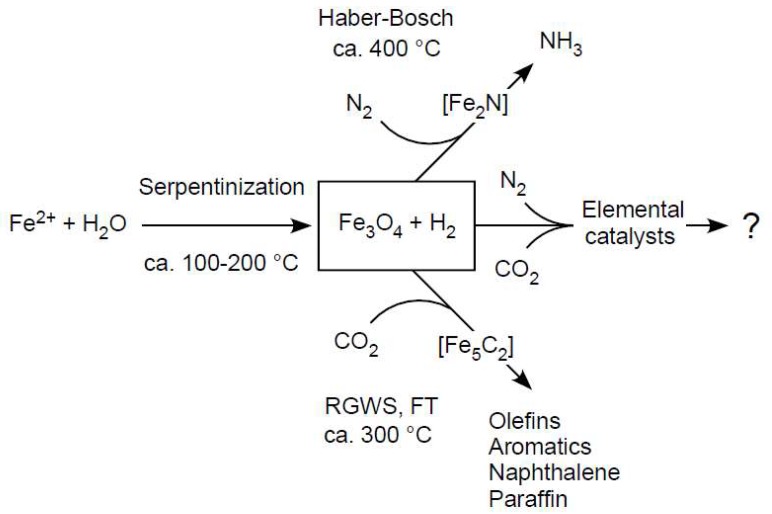
Possible connections between industrial processes and chemical evolution. See text. RWGS, reverse gas water shift reaction. FT, Fischer–Tropsch. The Haber–Bosch process starts with magnetite and generates nitrides [31,32]. The synthesis of gasoline from CO_2_ starts with magnetite and generates iron carbide, both catalysts appear to fulfill important but distinct roles [28]. Reaction parameters aimed at simultaneous reduction of N_2_ and CO_2_ are not well explored.

Magnetite is a common (although not ubiquitous) product of serpentinization. It is of interest in the context of CO_2_ reduction because it is the starting point to reach industrial catalysts for CO_2_ hydrogenation and for N_2_ reduction to ammonia via the Haber–Bosch process. Wei et al. [30] reported the H_2_-dependent reduction of CO_2_ to hydrocarbons (C_2_–C_11_), methane, and aromatics, with up to 22% CO_2_ conversion using Fe_3_O_4_ as the catalyst. During the reaction process, at about 320 °C and 30 bar (3 MPa), with H_2_/CO_2_ ratios of 1:1 to 6:1, Fe_3_O_4_ is converted by H_2_ in situ to iron carbide, Fe_5_C_2_, as analysis of the spent catalyst reveals. Fe_5_C_2_ is, in turn, thought to be the decisive catalyst for Fischer–Tropsch (FT) synthesis of longer hydrocarbons from CO, which is generated on Fe_3_O_4_ sites via a reverse water gas shift (WGS) reaction [30,99,100]. Note the involvement of WGS means that H_2_O is present during the reaction. FT and WGS reactions have long been discussed in the context of hydrothermal organic synthesis [101,102]. That magnetite itself is not an effective catalyst, as experimental studies reveal [68,103], is not the main point here. The point, as we see it, is that the effective catalyst, an iron carbide, is synthesized from CO_2_ and Fe_3_O_4_ in the presence of H_2_ during the reaction that mirrors conditions and chemical components found in serpentinizing hydrothermal vents, as outlined in Figure 2.

The participation of carbides in CO_2_ hydrogenation is well known from processes developed for industrial application. Metal–carbide interfaces catalyze CO_2_ conversion into CO [104] or methanol [105]. The literature on Fischer–Tropsch synthesis [106,107] shows that carbides are a product of reactions with iron catalysts. This happens especially with CO, because it decomposes to CO_2_ and chemisorbed carbon. The latter reacts further to produce iron carbides. As one can commonly observe magnetite, Fe_3_O_4_, in both fresh and spent iron catalysts, this compound is thought to be an active part of such reactions. Small amounts of native iron also seem to help the catalysis from CO to carbide [108]. Carbides have not been widely considered in an early evolution context, although they are also found in natural systems: Iron carbides are formed in the lower parts of the Earth’s crust (called ophiolite), but are uplifted over time—some of them even containing nickel [109,110,111]. In all of biology, there is only one carbide carbon known. Discovered in 2011 [28,29], it resides at one of the most crucial reactions for fueling ecosystems and in one of the most ancient enzymes known, nitrogenase.

Magnetite itself, without carbides and without exogenous H_2_, can reduce CO_2_ in H_2_O to acetate with roughly 3% conversion at 250 °C [112]. The greater high-temperature catalytic ability of reduced magnetite-containing carbides [106] or nitrides [32] and the lower-temperature (100–200 °C) catalytic ability of native metals [80] and awaruite [76] raise a question that needs to be spelled out clearly: Do serpentinizing hydrothermal systems improve their organic synthetic capacity during their lifespan by synthesizing better (more reduced) inorganic catalysts of organic reactions? That is, does serpentinization generate carbides and nitrides from magnetite and H_2_ in a similar manner to what chemical industry does? And if so, what kinds of products might one expect under conditions where both CO_2_ and N_2_ could be reduced simultaneously?

In a typical heterogeneously driven gas phase catalytic reaction, the first step of a catalytic reaction is adsorption of reactants on the surface of catalysts, followed by dissociation of reactants, product formation, and, finally, desorption of products from the surface of the catalyst. Heterogeneous catalysts naturally contain different types of catalytically active surface sites depending on their composition, crystallinity, particle size, and facet, and also impurities. If the catalyst contains several components (as the starting materials for the serpentinization process do) the surface composition might be different from a bulk structure. Moreover, the surface structure and composition of the catalyst can change dramatically during the catalytic reaction as it is the case for CO_2_ reduction over magnetite [30] and N_2_ hydrogenation to NH_3_ in the Haber–Bosch process [32].

This is potentially an interesting avenue of thought. The sequence of events underlying awaruite synthesis can, generally, be understood as large amounts of reduced iron and water giving rise to lesser amounts of H_2_, which then reduces divalent Ni and Fe in minerals to generate reduced magnetite and elemental metals Ni^0^ and Fe^0^ (Ni_3_Fe) which appear to have catalytic properties when it comes to H_2_-dependent CO_2_ reduction [76,80]. If so, the geochemical process is synthesizing catalysts for organic synthesis—constantly. As outlined above, processes of catalyst synthesis from Fe_3_O_4_ and H_2_ are demonstrably occurring in industrial applications for reduction of CO_2_ and N_2_.

## 7. Conserved Relicts in Metabolism: The Ni^0^ in CODH

**Figure 3:**
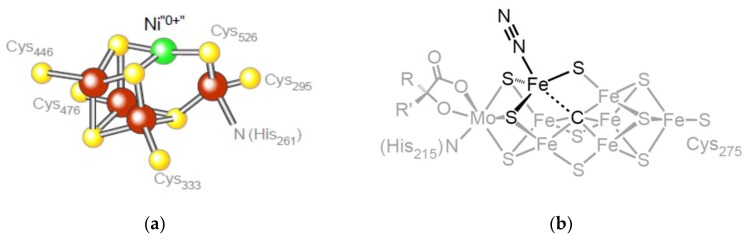
Relicts in metabolism. (**a**) The active site of CODH that interconverts CO_2_ and CO, redrawn from supplemental figure S5 in Ragsdale [26] underscoring the reduced Ni atom that binds CO_2_ in the proposed mechanism for the CO-generating reaction. (**b**) The carbide carbon in the active site of nitrogenase [28,29] and its proposed role in the catalytic mechanism [123].

The reaction mechanism of an ancient enzyme called carbon monoxide dehydrogenase (CODH) entails the generation of zero valent Ni (Ni^0^) as an intermediate in biological CO synthesis as shown in Figure 3a. CODH is involved in a pathway that is thought to be just as ancient (probably even more so) than the enzyme itself: The acetyl-CoA pathway. It is the only exergonic pathway of autotrophic carbon metabolism known [83,85,113]. Its exergonic nature allows acetogens and methanogens to generate transmembrane ion gradients in a process involving flavin-based electron bifurcation [114] during the process of CO_2_ fixation [81,115] and thereby conserve energy in the form of ATP via electron transfer phosphorylation (chemiosmosis).

Flavin-based electron bifurcation is a newly discovered mechanism of soluble (as opposed to membrane-associated) energy conservation [114,116,117]. Its principle is significant and we will encounter it again in a later section so it should be briefly explained here. The midpoint potential, *E*_0_^’^, of H_2_ (–414 mV) is not sufficiently negative to generate the low-potential reduced ferredoxin (*E*_0_^’^ = ca. –500 mV) that acetogens and methanogens require and use to reduce CO_2_ under physiological conditions. How, then, do acetogens and methanogens send electrons from H_2_ energetically uphill by roughly –100 mV? The electron pair from H_2_ is transferred to a flavoprotein, the flavin of which splits the pair: One electron goes energetically uphill to ferredoxin while the other goes energetically downhill to a more positive electron acceptor such as NAD^+^ (*E*_0_^’^ = ca. –320 mV) [115] or a heterodisulfide (*E*_0_^’^ = ca. –140 mV) [118] or similar [114]. The reduction of the downhill (more positive) acceptor energetically finances the reduction of the uphill acceptor so that ferredoxin is reduced and CO_2_ in the acetyl-CoA pathway can be fixed.

Although five of the six known pathways of autotrophic carbon metabolism also generate acetyl-CoA as the net end product of CO_2_ fixation starting with electrons from H_2_ [83] (the Calvin cycle generates glyceraldehyde 3-phosphate), only the acetyl-CoA pathway generates ATP from CO_2_ fixation. The other five require ATP input to fix CO_2_ [84]. That ATP input comes from an independent energy metabolism, typically aerobic or anaerobic respiration (sulfate reduction for example), that is independent of CO_2_ reduction. The reverse TCA cycle only requires the input of one ATP per CO_2_ [119,120], but it still requires ATP input. The acetyl-CoA pathway permits ATP synthesis from H_2_-dependent CO_2_ reduction. Why? It involves CO as an intermediate, a carbonyl moiety that becomes a carboxylate only after going through a sequence of metal carbonyl (CO–Ni), thioester (CO–S) and acyl phosphate (CO–OPO_3_^2–^) bonds, the latter of which phosphorylates ADP to generate the carboxylate. In all other pathways of CO_2_ fixation, CO_2_ is fixed as a carboxylate that subsequently has to be reduced, ultimately at the expense of ATP hydrolysis [83,84]. It is the zero valent Ni of CODH that generates CO for the acetyl-CoA pathway; that is the chemistry that makes the pathway exergonic [27,121,122].

## 8. Conserved Relicts in Metabolism: The Carbide in Nitrogenase

In terms of dry weight, life is 50% carbon and 10% nitrogen. Though CO_2_ can enter metabolism via six known carbon fixation pathways [83], there is only one known entry point for N_2_: Nitrogenase. It has long been known for its complex 7Fe-9S cluster harboring a light atom at its center and for existing in three related forms that differ with respect to the metal cofactor that is peripherally associated with the active site: Mo, V, or Fe [123,124,125]. It requires 16 ATP and 8 electrons per conversion of N_2_ into 2 NH_4_^+^ and it produces H_2_ as an unavoidable reaction by-product. The ATP is consumed by “archerases” in nitrogenase which hydrolyze ATP to induce conformational changes that alter the midpoint potential of 4Fe4S clusters to more negative values for N_2_ reduction [126].

In 2011, the light atom at the nitrogenase active site was identified as a carbon atom in the elemental state that is coordinated by four iron atoms, forming an essential Fe_4_C carbide at the catalytic heart of the enzyme [28,29] (Figure 3b). The carbide is generated during nitrogenase maturation from a methyl group donated to an active site Fe by S-adenosyl methionine (SAM) [127]. Synthetic analogs of the active site of nitrogenase even catalyze the synthesis of hydrocarbons from CO [128].

Nitrogenase is a very ancient enzyme that traces to the last universal common ancestor (LUCA) in ancestral genome reconstructions [87]. It has never been replaced during 4 billion years of evolution, nor has an alternative enzymatic mechanism been invented among microbes that would serve the same purpose of making N from N_2_ available for biosynthesis (amino acids, cofactors, nucleotides) and growth [129]. The incorporation of nitrogen into metabolism requires an iron carbide, as models for the nitrogenase catalytic mechanism suggest [130]. There is apparently no other way for biological systems to reduce N_2_ with the tools of enzymes and cofactors. That suggests to us that the carbide-containing active site of nitrogenase is a biological reconstruction of the naturally occurring inorganic catalyst that gave rise to organic N at the onset of biochemistry.

This inference parallels the situation with a native metal (Ni^0^) and CO synthesis in the exergonic acetyl-CoA pathway: There is apparently no mechanistic alternative to exergonic CO_2_ reduction with H_2_ that can be readily realized during 4 billion years of evolution. We suggest that elemental carbon (carbide) in nitrogenase and Ni^0^ in CODH represent relicts from the chemical environment that supported organic synthesis at life’s origin.

## 9. Weighing in on Caveats

One of the strengths of hydrothermal vent theories is that over the years since their first formulations, the gaps between spontaneous chemistry at vents and the chemistry of life have narrowed, not widened. Methane made abiogenically in substantial amounts at vents [22,52,71,72] stems from the same overall exergonic chemical reaction at the heart of methanogenesis in archaea, namely Equation (5). A similar case can be made for the acetogenic reaction [81,131].

4H_2_ + 2CO_2_ → CH_3_COOH + 2H_2_O (6)

Methanogens and acetogens are identified as the most ancient archaea and bacteria respectively, in reciprocally rooted trees for genes that trace to the last universal common ancestor (LUCA) [87,88]. Furthermore, H_2_-dependent methanogens appear as the most ancient archaea in some phylogenies [132] and clostridia (acetogens) appear as the most ancient bacteria, too [133], although lineage phylogenies among prokaryotes are always in flux and inferences from them are generally problematic because lateral gene transfer decouples physiology from phylogeny [134]. More direct observations are that methanogens abundantly inhabit vents [23,56] and the deep, subsurface oceanic crust [135,136] today.

But what about acetate or chemically reactive methyl groups like methanol or methyl sulfide? For example, the absence of more than nanomolar amounts of methyl sulfide was taken as evidence against the hydrothermal origins theory [137], but methanethiol is avidly assimilated by methanogens and acetogens in the acetyl-CoA pathway [83]. Similar reasoning applies to acetate, which is very scarce in hydrothermal effluents sampled so far [72], even though it readily accumulates in experiments intended to simulate vent conditions [80]. Acetate is the carbon and energy source for aceticlastic methanogens [81] belonging to the Methanosarcinales, which are abundant at hydrothermal vents [138,139], because the substrates they require for growth abound. The deep biosphere contains very large amounts of biomass [140,141] consisting of microorganisms that are generally starved for reducible carbon substrate, Heberling et al. [142] estimate 200 × 10^9^ tons of biomass in the marine igneous crust (that is, excluding sediment). That abiotic acetate or methylsulfide is scarce in vent effluents reported so far [56,137] might not directly reflect synthesis processes at depth, but microbial scavenging instead. Methane accumulates within vents because strong oxidants, which are typically lacking within vents, are required for methane oxidation [143].

Another criticism concerns synthesis. Miller and Bada [144] argued that FT type reactions readily catalyzed by native metals cannot generate organic compounds in hydrothermal systems because of catalyst inhibition by H_2_O and H_2_S. Holm et al. [52] explain, however, that off-axis hydrothermal systems of the Lost City type have low concentrations of H_2_S [145]. Additionally, awaruite can be formed at higher H_2_S activities (ca. 1 mmol/kg) than previously thought [54], and metals other than Fe, including Ni, are not inhibited by H_2_S.

Water activities are another issue typically raised by critics of hydrothermal vent theories [146]. Water hydrolyzes RNA and interferes with many reactions that generate specific prebiotic-type syntheses of particular organic molecules. This has spawned arguments that life must have arisen on land in dry or even desert-like conditions [147] or that essential reaction sequences giving rise to RNA monomers took place in the absence of water because that is how they work best in the laboratory with energy from *UV* light [12]. Nobody has yet proposed that RNA replication took place without water. Life is 80% water by weight, about 60% protein and 25% RNA by dry weight [148], with protein synthesis consuming about 75% of a cell’s energy budget [149]; life counters hydrolysis problems by synthesizing polymers faster than they are hydrolyzed, at the expense of chemical energy.

An underappreciated aspect concerning water activity is that serpentinization takes place in rock that is initially very dry and that roughly 12 to 37 mol water is consumed per mol H_2_ produced (reactions **1**–**4**). Placed in the context of methane or acetate synthesis (reactions **5–6**), roughly 100 mol of H_2_O is consumed per CO_2_ or carbonate that is reduced to the level of a methyl group. Furthermore, a great deal of additional water is simply converted into hydroxylated minerals during serpentinization. At the same time that water is being consumed, reactive gasses (H_2_, CO) are being generated. Gas reactions on solid phase catalysts in serpentinization studies should not be neglected, nor should the circumstances that hydrophobic organics synthesized at hydrothermal vents will undergo phase separation and generate molecular environments of low water activity, which is the main function that enzymes provide in catalysis: Exclusion of water from the active site [150]. The crust hosting hydrothermal systems is necessarily replete with localities of low water activity and water disappears where reducing power for carbon reduction is generated.

## 10. Do Genomes Help?

Recent work has examined the origins problem from a novel top-down perspective. Standard approaches analyzed genomes to see which genes are universal to all cells, recovering about 30 genes involved in information processing [88]. Weiss et al. [87] constructed all trees for all proteins from 2000 genomes and identified proteins that are not universal to all cells but that are ancient by phylogenetic criteria. They looked for genes that trace to the LUCA because they are present in bacteria and archaea, but present by virtue of vertical inheritance from LUCA as opposed to late lateral gene transfer [87,88]. Based upon the kinds of genes that trace to LUCA, the results indicated that LUCA lived from dissolved gasses: H_2_, CO_2_, CO, N_2,_ and H_2_S. The results indicated that for carbon assimilation, LUCA used the simplest and most ancient of the six known pathways of CO_2_ fixation, the acetyl-CoA pathway germane to methanogenesis and acetogenesis, but it lacked the methyl synthesis branch suggesting that it relied on a geochemical supply of methyl groups. LUCA had nitrogenase, it had no traces of light utilization, its environment was hot, contained H_2_, CO_2_, CO, N_2_, H_2_S, and metals—an environment that looks very much like a hydrothermal vent. Genomes trace LUCA to a site of rock–water–carbon interactions.

LUCA was able to harness ion gradients via the rotor-stator ATPase, but proteins of the ion gradient generation (pumping) were missing [87], which would have been possible, if LUCA lived at the vent of a serpentinizing system. There, geochemically generated ion gradients (alkaline inside versus the more neutral ocean) could be harnessed before the machinery was invented that allows cells to pump with the help of an ion gradient generated by a chemistry that is specified by genes [24,131]. In short, LUCA was half-alive, dependent upon geochemical organic synthesis and the chemical disequilibrium of its environment to harness carbon and energy. That might sound radical, but no theory for origins can operate without some kind of sustained chemical synthesis from the environment. A strong argument for origins at vents is the congruence between the basic CO_2_ reduction reactions of serpentinization and the chemistry of acetogen and methanogen metabolism [24,86]. Metabolic pathway reconstructions of phosphate independent reactions uncovered the essential role of ferredoxin-dependent redox reaction and thioesters in ancient anabolic biochemistry [151,152]. LUCA’s metabolism and genetic code were heavily dependent upon methyl groups [87].

The kind of LUCA that emerges from genomic reconstruction would have starved (low H_2_ and CO_2_ activities) and perished (*UV* light) at the surface—what it needed to survive was provided by rock–water interactions in the crust: Reactive gasses. Perhaps the crust was the first environment on Earth to have been inhabited [4,55]. Biologists and chemists have long held that inorganic catalysts/electron donors like FeS centers preceded organic catalysts/electron donors like NADH in evolution [153,154]. Similarly conserved base modifications (methylations, sulfur additions) in tRNA, in particular in the anticodon loop that allows the modern genetic code to operate, reflect the chemical environment within which the genetic code arose [87,88].

LUCA also harbored hydrogenases [87]. In Figure 3, the active sites of CODH and nitrogenase underscore the role of Ni^0^ and a carbide in the entry of carbon (from CO_2_) and nitrogen (from N_2_) into the biosphere. These active sites represent bottlenecks in the origins and early evolution of metabolism. For the reduction of carbon and nitrogen, electrons stemming from H_2_ as the proximal carrier were required. But in metabolism, H_2_ never interacts directly with C or N, rather it enters metabolism via metal atoms in hydrogenases. Electrons from H_2_ always enter metabolism via metals in the active site of hydrogenase or hydrogenase domains. There are three different kinds of hydrogenases known, they are phylogenetically unrelated and have very different active sites: The [NiFe] hydrogenase, the [FeFe] hydrogenase, and the [Fe] hydrogenase, the latter lacking FeS clusters [155]. Common to all three active sites, however, is that H_2_ relinquishes its electrons via interaction with an Fe atom that is coordinated by one or more CO molecules [156]: Iron(II) carbonyls participate in H_2_ activation in three independently arisen hydrogenase active sites. In contrast to CODH and nitrogenase, hydrogenases have appeared three times independently in evolution, but the iron carbonyl catalyst is conserved, indicating that the catalyst is older than the proteins that harbor it.

## 11. What Next?

What has been missing from hydrothermal vent-based research on origins is a decisive experiment where a wide variety of amino acids and bases arise under genuinely realistic conditions, although an early attempt was made by Hennet et al. [34]. FeS generally does not efficiently convert CO_2_ into reduced carbon, either in the laboratory or in metabolism. Native metals do [80], and they furthermore generate the biologically relevant carbon species that occur at the core of metabolism: Formate, acetate, pyruvate, and methyl groups. The reason that FeS does not work well, but Fe^0^ does, stated most simply, is that CO_2_ reduction in biology (and perhaps more generally) is always a two-electron reaction, whereas FeS only undergoes one-electron reactions. Wächtershäuser has stressed the role of FeS in primordial biochemistry [157] and reported extensive experiments using FeS as a catalyst in reactions involving CO as a reductant, in which amino acids were observed [158]. Bases (heterocycles) have not been observed so far in FeS catalyzed reactions, nor has CO_2_ reduction using FeS catalysts ever been convincingly demonstrated in terms of reaction rates or product yields so far. CO_2_ reduction using H_2_ and Ni_3_Fe catalysis at higher temperatures (200–400 °C) produce good yields of methane [76], but methane is rather an end product of metabolism than a building block of life.

**Figure 4:**
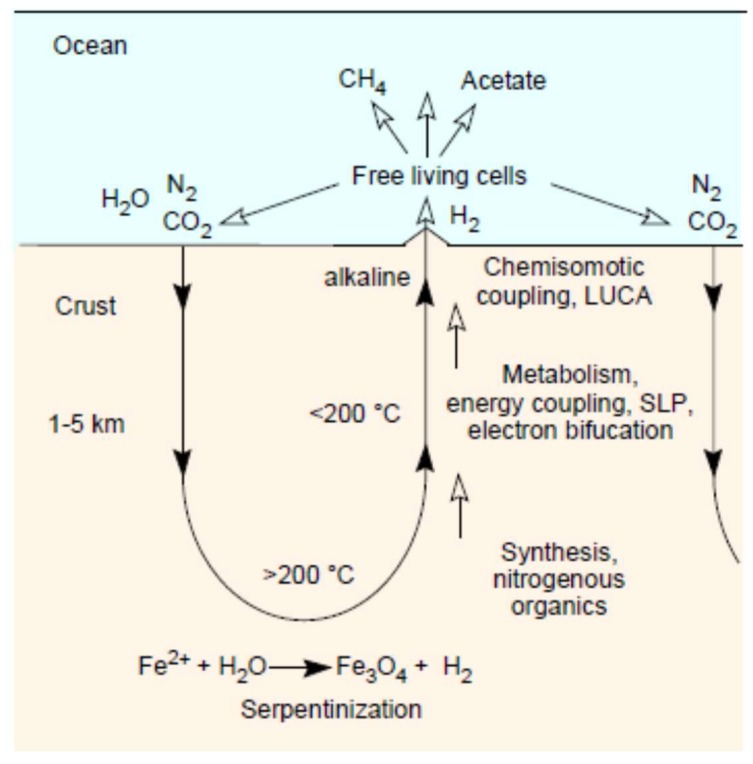
Possible processes at depth and at the ocean floor in serpentinizing systems. See text.

**Figure 5:**
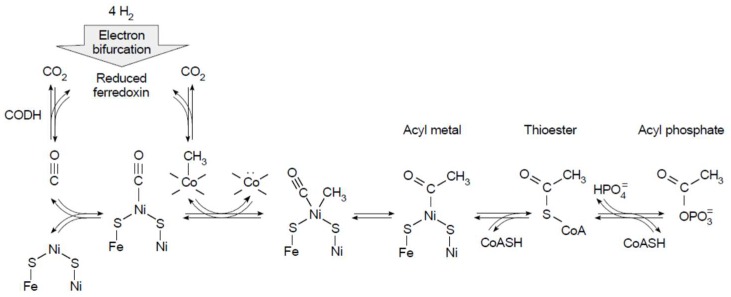
An ancient pathway. The diagram summarizes the biological energy conservation from ferredoxin to acyl phosphate in the acetyl-CoA pathway in an early evolution context [23,78,112]. Note that the reactions shown also occur without enzymes under suitable conditions [152]. See text. For an explanation of electron bifurcation see [106]. CODH: Carbon monoxide dehydrogenase.

That is a complicated way of saying that work investigating the role of hydrothermal chemistry in an origins context should possibly be looking at higher temperatures, higher pressures, low water activities, and catalysts containing native metals, carbides, and minerals like magnetite. It is possible that efficient synthesis of amino acids and bases from CO_2_ and N_2_ requires conditions where both gases are reduced to an appreciable extent simultaneously. This does not mean that Haber–Bosch-like conditions (500 °C) need to be employed for prebiotic type synthesis, because there is no imperative to generate high yields or specificity from such experiments. It does, however, suggest that FeS, despite its unassailably essential role in the physiology of life, despite its unquestionable antiquity [153], and despite its unquestioned ability to generate thioesters from CH_3_SH and CO [159] might not be the right electron donor to get CO_2_ reduced in a hydrothermal context. Although FeS is crucial to make iron biologically accessible, and sulfur compounds were probably very important for the transition from thermal to chemical activation, catalysts that promote two-electron reactions fit better to the chemistry of carbon. We also need to consider the very real possibility that some reactions work better (or at all) at greater depth, products being transported by hydrothermal current to cooler environments near the surface where they can react under milder conditions (Figure 4), and where lower temperature reactions more similar to energetically coupled metabolism (as opposed to synthesis), such as those reported by Muchowska et al. [160] (although under acidic conditions) and Varma et al. [80], or oscillating thioester reactions such as those reported by Semenov et al. [161], come into play. Even at hydrothermal vents, in terms of catalysis, some reactions take place on certain surfaces at higher temperatures, others will only function at lower temperatures with different catalysts. In that sense, the sequence of reactions in the acetyl-CoA pathway [83] from electron bifurcation, to CO, to acyl metal bonds, to thioesters that yield acyl phosphates (Figure 5), which phosphorylate ADP to generate ATP, can be seen as the spatially condensed and enzymatically catalyzed version of a spontaneous geochemical process, the thermodynamic drive for which stems from the natural tendency of CO_2_ to be reduced with electrons from H_2_. That is, similar to the iron(II) carbonyl of hydrogenases, the chemical reactions of the acetyl-CoA pathway themselves are older than the enzymes that catalyze them.

Early papers on vents and life [1] voiced similar considerations about the possibility of different processes occurring at different depths. This became more evident as it became clear that serpentinization was relevant to synthesis at vent and origins [50]. A spectrum of processes across depths and conditions needs to be considered. And why, one might ask, have industrial chemists not already exhaustively explored the kinds of reactions sketched at the right of Figure 2? Reacting H_2_, N_2_ and CO_2_ on Fe_3_O_4_ at temperatures needed for nitrogen reduction is not only Haber–Bosch expensive (energy intense), it is not going to generate any specific product. Chemists have not yet explored all kinds of conditions that might be interesting for prebiotic chemistry [162], the high-pressure–high-temperature reaction parameter space holds potential for investigation. Hydrothermal vents have been supporting microbial life for 3.5 billion years [163], perhaps because it arose there.

There also might be other parallels between bio- and geochemistry. For example, the formation of Ni_3_Fe is still a mystery, and it poses an energetic problem similar to flavin-based electron bifurcation: The electrons from H_2_ need to go energetically (far) uphill to reduce Fe^2+^ to Fe^0^ under the conditions in hydrothermal systems. Might there be a geochemical redox process akin to biological electron bifurcation, that is, might the reduction of Fe^2+^ to Fe^0^ via hydrogen be energetically coupled to (and financed by) Ni^0^ deposition? It would help to explain why native Ni and Fe are deposited together, and in the case of awaruite in a stoichiometry favoring Ni, even though it is much rarer than Fe in serpentinizing systems.

**Figure 6:**
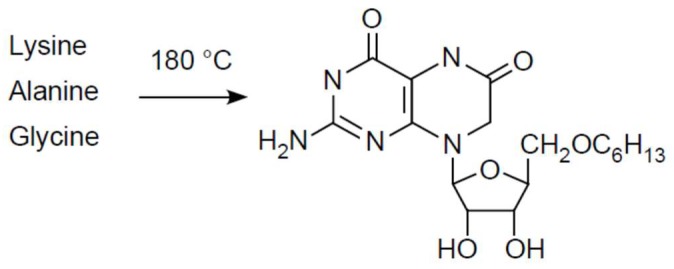
Pterin riboside from amino acids. One of the products obtained by Heinz et al. [165] without catalysts from dry heating of three amino acids is shown. Pterins are important cofactors in the acetyl-CoA pathway [83,86,166]. Note the *N*-glycosidic bond of the heterocyclic to ribose.

Some of the organic compounds found on carbonaceous chondrites are apparently formed via FT type reactions [164]. FT type reactions in the presence of NH_3_ generate the familiar nucleobases, but at very low conversions (ca. 0.02% of product) and requiring short heating up to ca. 600 °C [164]. An old experiment [165] serves as a reminder: Heating three amino acids (combinations of glycine, alanine, valine, lysine, asparagine and glutamine) without water to temperatures between 160 °C to 200 °C for 4 to 6 h or to temperatures between 180 °C to 350 °C for 1 to 2 h produces, inter alia, the compound shown in Figure 6.

There has to be something innately natural to the chemistry of life that its chemical constituents will assemble effortlessly when the right conditions and catalysts are found. Perhaps reducing CO_2_ and N_2_ under chemical industry conditions milder than Haber–Bosch, with catalysts derived from H_2_ and Fe_3_O_4_, will deliver a hydrothermal version of the Miller–Urey experiment, with a diversity of relevant nitrogenous compounds. This is an experiment that hydrothermal theories have been missing. The surface structure, morphology, facets, and impurities of catalysts should be also taken into account as crucial parameters. Concomitant reduction of N_2_ and CO_2_ might yield a diversity of compounds relevant to life more readily than adding NH_3_ to reduced carbon compounds. Regardless, by better understanding the sequence of chemical events within hydrothermal systems we should gain insights into catalysts that can further close the gap between rock–water–carbon interaction in vents, and life.
